# Tuning and Fine-Tuning of Synapses with Adenosine

**DOI:** 10.2174/157015909789152128

**Published:** 2009-09

**Authors:** A.M Sebastião, J.A Ribeiro

**Affiliations:** Institute of Pharmacology and Neurosciences, Faculty of Medicine and Unit of Neurosciences, Institute of Molecular Medicine, University of Lisbon, Lisboa, Portugal

**Keywords:** Adenosine, receptor cross-talk, metamodulation, neurodegenerative diseases, epilepsy, G protein coupled receptors, ionotropic receptors, receptor kinases.

## Abstract

The ‘omnipresence’ of adenosine in all nervous system cells (neurons and glia) together with the intensive release of adenosine following insults, makes adenosine as a sort of ‘maestro’ of synapses leading to the homeostatic coordination of brain function. Besides direct actions of adenosine on the neurosecretory mechanisms, where adenosine operates *to tune *neurotransmitter release, receptor-receptor interactions as well as interplays between adenosine receptors and transporters occur as part of the adenosine’s attempt *to fine tuning *synaptic transmission. This review will focus on the different ways adenosine can use to trigger or brake the action of several neurotransmitters and neuromodulators. Adenosine receptors cross talk with other G protein coupled receptors (GPCRs), with ionotropic receptors and with receptor kinases. Most of these interactions occur through A2A receptors, which in spite their low density in some brain areas, such as the hippocampus, may function as metamodulators. Tonic adenosine A2A receptor activity is a required step to allow synaptic actions of neurotrophic factors, namely upon synaptic transmission at both pre- and post-synaptic level as well as upon synaptic plasticity and neuronal survival. The implications of these interactions in normal brain functioning and in neurologic and psychiatric dysfunction will be discussed.

## INTRODUCTION

The concept of neuromodulation evolved from that of neurotransmission, and was initially identified as mechanism where an endogenous substance, released either from the pre- or the post-synaptic component, influences the release (pre-synaptic modulation) or the action (post-synaptic modulation) of the neurotransmitter. As our understanding of the structure and functioning of a synapse advanced, this concept has gained complexity. We now know that several modulatory substances are present at a given synapse, being released not only from neurones but also from glia. Astrocytes sense and respond to neuronal activity and are actively involved in signal transmission [133]. Variations in gliotransmission may add to dysfunctions of neurotransmission and contribute to disorders of the nervous system [77]. So, each neuromodulator has many different possibilities to fine tune neuronal activity. Among the diversity of neuromodulators in the brain, adenosine and/or ATP are key fine-tuners since: 1) they are among the most relevant players in neuron-glia communication [63], 2) they can affect the release and the action of many neurotransmitters and neuromodulators, and 3) they are released by almost all cells. 

Remarkably, the discovery of the presynaptic inhibitory action of adenosine [69] started with two unexpected findings in experiments carried out at a single synapse model, the neuromuscular junction. The mobile of the study was unrelated to adenosine research and the main aim was to evaluate the influence of cyclic AMP upon neurotransmitter release. Adenosine was merely used as a tool known to increase cyclic AMP in the nervous system and, if anything, it was expected to increase transmitter release, but decreased it! Also unexpectedly, the inhibitory action of adenosine was prevented by theophylline, at the time well known as a phosphodiesterase inhibitor, therefore expected to amplify cyclic AMP mediated events [70]. These pioneer observations gave rise to a whole line of research on the neuromodulatory role of adenosine, and introduced the idea that adenosine could be a relevant molecule to make the tuning of synapses, in other words, to modulate neurosecretory mechanisms at nerve terminals. This idea was soon reinforced by the finding that ATP is released together with acetylcholine [145] and mimics the adenosine effect [125, 126]. At the synaptic cleft, ATP is degraded by a cascade of ectoenzymes [173], being a relevant source of extracellular adenosine [123]. 

It took nearly 2 decades to recognize that the same nerve terminal can possess both inhibitory (A1) and excitatory (A2A) adenosine receptors [30]. Research on neuromodulatory actions of A2A receptors suffered from the initial prejudice that these receptors were only expressed in the striatopallidal GABAergic neurones and olfactory bulb [4, 132]. First evidence that the A2A adenosine receptor could influence neuronal communication in other brain areas than the striatum or olfactory bulb appeared in a study using hippocampal slices [140]. This was followed by evidence that A2A mRNA and A2A receptor protein are expressed in the hippocampus [36]. The initial scepticism was broken [141] and it is now widely recognized that A2A receptors are expressed in several brain regions though in lower levels than the striatum. 

We now know that adenosine possesses different ways to trigger or brake the action of several neuromodulators. The role of A2A receptors in the brain might be less related to a direct modulation of neuronal activity, but instead, to their ability to interact with receptors for other neuromodulators or neurotransmitters, there is to say, to fine tune neuronal activity. In this review we will discuss particularly this new established concept [135], which is becoming more and more extensive to the multiplicity of neurotransmitter receptors with which adenosine interacts.

## 1. INTERACTIONS WITH G-PROTEIN COUPLED RECEPTORS

Adenosine receptors are G-protein coupled receptors (GPCRs) and it is not difficult to envisage the different possibilities they may use to interact with other GPCRs, if co-expressed at the same cell. Dimerization of GPCRs, either homo- or heteromerization has been accepted since the strong evidence that GABAB receptors are dimers of two GABAB receptor molecules [158]. First hints of A2A/D2 heterodimers in the striatum were, however, several years a head [60] and their functional relevance is now firmly established [57]. Futhermore, GPCRs most frequently share α subunits, not to speak of the βγ subunits, which are common to different G–proteins and may change activation equilibrium of other GPCRs with its own G-protein. Last, but not least, there are many possibilities of cross-talk with related transduction pathways, which have several kinases and other key molecules in common.

The ubiquitous nature of adenosine, that is to say, its presence and release from almost all cells, together with a broad distribution of adenosine receptors throughout the brain [124] puts it into a privileged position to behave as modulator of neuromodulators, as compared with other GPCR ligands with more restrict brain location. 

### 1.1. Dopamine Receptors

A first indication that A2A receptors could interact with dopamine D2 receptors came from binding studies showing that activation of A2A receptors decreases the affinity of dopamine D2 receptors in rat striatal membranes [60]. This A2A/D2 interaction seems to be essential for the behavioural effects of adenosine agonists and antagonists, like caffeine [56]. The interest on the A2A/D2 interaction, quickly expanded to psychiatry and neurology fields such as, schizophrenia and Parkinson’s disease, and has been matter of many reviews by groups that have been implicated in the subject since its origin [57, 67]. Very briefly, implications for Parkinson’s disease mostly reside on the fact that A2A receptors counteract D2 receptor activation. A2A receptor antagonists entered clinical trials and although the benefit was not as much as expected, a decrease in dyskinesia and a slight decrease in the dose of L-DOPA required to attain therapeutic benefit has bee found in patients under A2A antagonism co-therapy [see 104]. For schizophrenia, the therapeutic potential is for the A2A receptor agonists, rather than antagonists, exploring the benefit of the negative interaction between A2A receptors and D2 receptors [57, 67]. 

A1 and D1 adenosine receptors also interact in the basal ganglia [59] an interaction that probably occurs at the functional level and has implications for the control of GABA release at the substancia nigra [64] and dopamine release in the striatum [111]. Furthermore, A1 receptor activation has been shown to facilitate D1 receptor desensitization [93].

### 1.2. Neuropeptides

By activating A2A receptors, adenosine tonically potentiates a facilitatory action of the neuropeptide calcitonin gene-related peptide (CGRP) on neurotransmitter release from motor nerve terminals [26]. The ability of CGRP to facilitate synaptic transmission in the CA1 area of the hippocampus is also under tight control by adenosine; thus, tonic A1 receptor activation by endogenous adenosine 'brakes' the action of CGRP, while the A2A receptors trigger it [138]. If it also applies to other areas of the nervous system, this A1 receptor mediated inhibition of the action of CGRP, together with the A1-induced inhibition of CGRP release [16] can be related to pain inhibition by adenosine. Indeed, CGRP, is a potent vasodilator released from the activated trigeminal sensory nerves, dilates intracranial blood vessels and transmits vascular nociception, being implicated in the genesis of vascular pain such as migraine. Hence, inhibition of trigeminal CGRP release or CGRP receptor blockade have been proposed as promising anti-migraine strategies [71]. 

The pain control by adenosine involves, however multiple mechanisms, and its discussion is clearly outside the scope of the present review. The reader may refer to recent reviews covering this subject [130, 136]. Just briefly, adenosine has anti-nociceptive actions through A1 receptors, but A2A receptors also contribute to reduce inflammatory pain by operating anti-inflammatory mechanisms. This led to an increasing interest in the development of drugs that, by influencing extracellular adenosine levels, could have analgesic actions. Promising examples are the inhibitors of adenosine kinase, which enhance extracellular adenosine levels by reducing its intracellular phosphorylation into AMP. As noted more than a decade ago, adenosine kinase inhibitors have anti-nociceptive properties [86]. Devices that promote local delivery of adenosine would overcome toxicity of adenosine kinase inhibitors, as well as systemic side effects of adenosine A1 receptor agonists, and may prove useful for idiopatic pain control in a way similar to that proposed for epilepsy control [11].

The facilitatory action of the vasoactive intestinal peptide (VIP) upon ACh release from motor nerve endings is triggered by adenosine, which accumulates extracellularly during high frequency stimulation and activates A2A receptors [29]. VIP also enhances synaptic transmission at the CA1 area of the hippocampus, and this is due to an enhancement of inhibition of inhibitory interneurones, therefore reducing inhibitory input to pyramidal glutamatergic neurones [38, 40]. This action of VIP is dependent on both A1 and A2A receptor activation by endogenous adenosine [37, 39] since it is blocked or markedly attenuated by antagonists of A1 as well as of A2A adenosine receptors. Interestingly, the finding that VIP-induced modulation of GABA release from hippocampal nerve terminals is under control of adenosine A1 receptors constitutes one of the first evidences for a role of A1 receptors in mature hippocampal GABAergic terminals. A1 receptors can directly inhibit GABA release in immature hippocampal neurons [82] but this action is lost in mature GABAergic neurons [91, 169]. 

Neuropeptide Y (NPY) agonists inhibit presynaptic calcium influx through N and P/Q type calcium channels and inhibit glutamate release at the CA3-CA1 synapse of rat hippocampus, an action that is fully occluded by co-activation of adenosine A1 receptors [119]. Interestingly, the inhibitory action of the GABAB agonist, baclofen was not occluded by adenosine receptor activation, indicating that the cross talk between A1 receptors and NPY receptors is not extensive to other GPCRs such as the GABAB receptor. Adenosine/NPY interaction can in some cases occur behind receptor interaction, namely at the release level. Thus, exocytosis of NPY containing vesicles is facilitated by A2A receptor activation in PC12 cells [105], but this does not occur in nerve endings from the rat mesenteric artery, where adenosine receptors affect noradrenaline but not NPY release [51].

In cultured primary hippocampal neurones δ-opioid and cannabinoid CB1 agonists act synergistically to activate PKA signalling through Gi-β/γ dimmers, and this synergy requires A2A receptor activation [167]. CB1 agonists also act synergistically with μ-opiate receptors in primary nucleus accumbens/striatal neurones and again this synergy requires adenosine A2A receptors [168]. Moreover, modifications in the expression of several types of opioid receptors was recently detected in mice lacking the A2A AR gene [6], suggestive of a functional interplay between adenosine A2A receptors and opioid receptors. An increase in adenosine levels in the cerebrospinal fluid has been detected in humans following intrathecal administration of morphine [54], showing that interactions between opioids and adenosine also occur beyond the receptor cross-talk level. 

Relevant consequences of the interactions between adenosine and opioids are pain control and drug addiction. Remarkably, in neuropatic rats the morphine-induced adenosine release is reduced [129]. Since morphine-induced adenosine release may contribute to pain control, due to the antinociceptive actions of A1 receptor activation, a decrease in adenosine release in neuropatic rats may explain the decreased efficacy and potency of opioids in the treatment of neuropatic pain. This, again, points towards the interest of adenosine augmentation strategies for the control of idiopatic pain. As regards heroin addiction, A2A receptor blockade eliminates heroin-seeking behaviour in addicted rats, suggesting that A2A receptor antagonists may be effective therapeutic agents in the management of abstinent heroin addicts [168]. The mechanisms wherein A2A receptor antagonists attenuate drug addicted behaviours most likely involve a complex network of receptors and neuronal circuits, that includes not only opiate receptors but also other GPCRs, such as cannabinoid CB1 receptors and dopamine D2 receptors (see below).

**Fig. (1):**
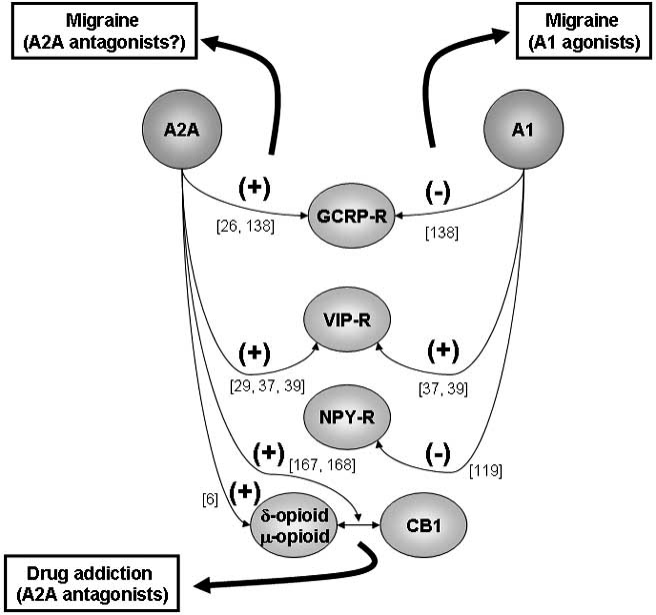
**Schematic representation of the reported cross-talk between adenosine receptors and receptors for neuropeptides.** The putative therapeutic value of those interactions is also indicated. (+) denotes facilitation and (-) denotes inhibition. See text and references (indicated in brackets) for details.

In summary (Fig.**1**), A2A receptor activation facilitates the action of neuropeptides, such as CGRP, VIP and opioid receptors. The pattern is less constant for A1 receptors, which in some cases inhibit neuropeptide actions, as it has been shown for CGRP and NPY, and in other cases are required to allow neuropeptide action, such as for VIP. Reciprocal interactions at the release level, with peptides inducing release of adenosine and adenosine, through A2A receptor activation, facilitating peptide release, also occur, but these are typical neuromoulatory actions and occur behind the receptor cross-talk level.

### 1.3. Metabotropic Glutamate Receptors

Metabotropic glutamate receptors (mGluRs) encompass a family of receptors which negatively couple to adenylate cyclase (Group II: mGluR2, mGluR3; Group III: mGluR4, mGluR6, mGluR7, mGluR8) or positively couple to phospholipase C (PLC) signalling (Group I: mGluR1 and mGluR5) Furthermore, activation of metabotropic glutamate receptors may potentiate cAMP responses mediated by several receptors positively coupled to adenylate cyclase, namely, A2 adenosine receptors, VIP receptors, and β -adrenergic receptors [3, 162]. 

mGluRs, most probably through PLC/PKC signalling, influence and A1 adenosine receptor functioning in neurones [see 136]. Agonists of Group I mGluRs also attenuate GABAB mediated inhibition on synaptic transmission, a process that involves PKC activity [143]. In addition, activation of PKC by phorbol esters or activation of PKC-coupled metabotropic glutamate receptors suppress the inhibitory action of A1 receptor agonists on glutamate release from cerebrocortical synaptosomes [14]. 

The inhibitory effects of an adenosine A1 receptor agonist and of agonists of Group II mGluRs are less than additive [45] most probably due to sharing of common (Gi/o) G proteins [172]. 

Activation of A3 receptors leads, through a PKC-dependent process, to a marked attenuation of the presynaptic inhibitory functions of cAMP-coupled metabotropic glutamate receptors at the CA1 area of the hippocampus [101]. The action of PKC and probably also that of A3 receptors on metabotropic glutamate receptors might result from an inhibition of the coupling of metabotropic glutamate receptors with Gi/o proteins [101]. Thus, the actions of adenosine A1 or A3 receptors and those of metabotropic glutamate receptors in neurones are mutually occlusive, through a process probably involving the cross-talk of transducing systems, namely PKC-induced changes in G-protein signalling, or the sharing of G-proteins, as proposed several years ago to explain the mutual occlusion between presynaptic adenosine A1 and α_2_-adenergic receptors [98].****

In contrast, in cultured astrocytes, activation of A1 receptors enhances the intracellular calcium responses induced by mGluR5 activation [25, 109], a process that involves a pertussis toxin sensitive G protein, therefore Gi or Go [25, 155]. Interestingly, besides the synergy with mGluRs, adenosine-induced calcium responses in astrocytes also require A1/A2 receptor cooperation and enhancement of cAMP levels [108]. 

As regards A2A receptor agonists, they act synergistically with agonists of Group I mGluRs to modulate dopamine D2 receptors in the rat striatum, decreasing the affinity state of these receptors [58]. Furthermore, A2A receptors act synergistically with mGlu5 receptors to increase DARPP-32 phosphorylation, so that blockade of one of the receptors is enough to prevent phosphorylation induced by activation of the other receptor [107]. A2A and mGlu5 receptors are colocalized postsynapticaly with D2 receptors in medium spiny neurons at the striatum, inhibiting D2 receptor functioning in a synergistic way [104]. They also co-localize presynaptically at striatal glutamatergic terminals where they facilitate glutamate release in a synergistic manner [116, 127]. Prevention of mGlu5 and A2A synergy eventually at the pre- and the post-synaptic level will therefore lead to decreased glutamate release, with consequent reduced excitotoxicity, together with a facilitation of D2 dopaminergic receptor functioning, and this is the rational for the use of antagonists of these receptors as anti-Parkinsonian drugs. Indeed simultaneous blockade of A2A and mGlu5 receptors had high efficacy to reverse Parkinsonian deficits in rodents [24, 84]. Combined antagonism of glutamate mGlu5 and adenosine A2A receptors also efficiently reduced alcohol self-administration and alcohol-seeking in rats [2], further reinforcing the importance of the mGlu5 and A2A receptor interaction in the mesolimbic and basal ganglia areas. 

**Fig. (2):**
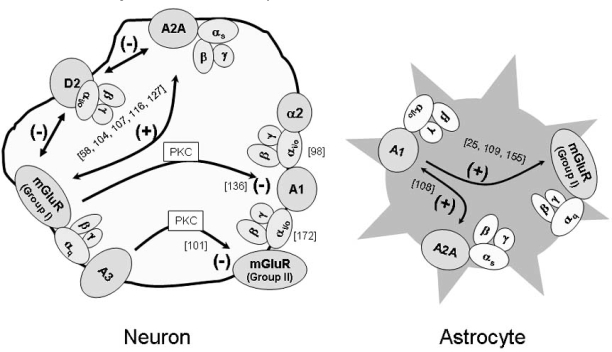
**Schematic representation of the reported cross-talk between adenosine receptors and metabotropic glutamate receptors.** The coupling of each receptor to G proteins (αβγ subunits) is also indicated. (+) denotes facilitation and (-) denotes inhibition. Whenever the mechanisms involved in the interaction have been evaluated, they are indicated close to the arrow. PKC: protein kinase C. See text and references (indicated in brackets) for details.

In summary (Fig. 2), one of the most promising interactions between adenosine receptors and mGluRs is the synergy between A2A receptors and mGluR5, which reflects into an attenuation of D2 receptor mediated actions. Blockade of A2A receptors as well as of mGluR5 may therefore allow enhanced dopaminergic function in the basal ganglia, with implications for Parkinson’s disease and drug addiction therapies. The synergy between adenosine receptors and mGluRs to enhance calcium signalling in astrocytes may enhance neuron/glia interactions but its consequences in disease states are yet to be evaluated. 

### 1.4. Cannabinoid Receptors

The high density of adenosine A2A receptors in the basal ganglia, together with the profound motor depressant effects of cannabinoids, prompted the interest of investigating a putative cross talk between A2A and CB1 receptors in this brain area. CB1 receptor signalling in a human neuroblastoma cell line is dependent on A2A receptor activation [15]; furthermore, blockade of A2A receptors counteract the motor depressant effects produced by CB1 receptor activation in vivo [15], suggesting that A2A receptor activation facilitates CB1 receptor function in the basal ganglia. Interestingly, the motor depressant effect produced CB1 receptor activation is attenuated by genetic inactivation of DARPP-32 [5], which is abundantly expressed in the medium spiny neurons of the striatum and is crucially involved in the striatal actions of cyclic AMP coupled receptors [76], as it is the case of A2A receptors. It thus appears that A2A receptors, through cAMP production and DARPP-32 activation are key molecules to enhance CB1 receptor activity in basal ganglia. Striatal A2A and CB1 receptors may also directly interact at the molecular level since CB1 and adenosine A2A receptors form heteromeric complexes once transfected to HEK-293T cells [15]. 

A2A receptor activation is required for the synergistic actions between CB1 receptors and μ-opioid receptors in NAc/striatal neurons [168], as well as for the synergistic actions that occur between CB1 agonists and D2 agonists [167]. Since both CB1 and D2 receptors couple to Gi proteins, their activation is expected to decrease cAMP production. However, when co-activated, these receptors may facilitate cAMP mediated signalling and this involves βγ dimmers of Gi proteins [167]. There is also a synergy between CB1 receptor agonists and ethanol. In all instances, synergy requires activation of adenosine A2A receptors [167]. 

A significant reduction of tetrahydrocannabinol-induced rewarding and aversive effects was found in mice lacking A2A adenosine receptors, indicating a specific involvement of A2A receptors in the addictive-related properties of cannabinoids [147]. Somatic manifestations of tetrahydrocannabinol withdrawal were also significantly attenuated in A2A receptor knockout mice, but antinociception, hypolocomotion and hypothermia induced by acute tetrahydrocannabinol administration, remained unaffected [147]. 

**Fig. (3):**
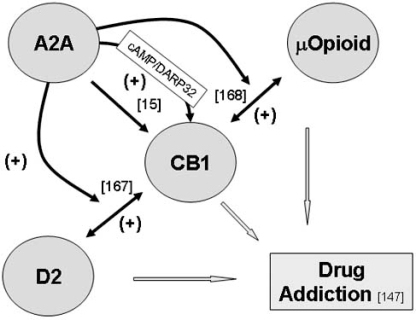
**Schematic representation of the reported cross-talk between adenosine A2A receptors and CB1 receptors and corresponding implications for drug addiction.** (+) denotes facilitation and (-) denotes inhibition. Whenever the mechanisms involved in the interaction have been evaluated, they are indicated close to the arrow. cAMP: cyclic AMP; DARPP-32: Dopamine- and cAMPregulated phosphoprotein. See text and references (indicated in brackets) for details.

In summary, the above mentioned data suggests that adenosine A2A receptors facilitate CB1 receptor signalling as well as the interplay between CB1 receptors and other key receptors and pathways involved in drug addiction (Fig. 3). Surprisingly, however, chronic caffeine consumption, therefore chronic blockade of adenosine receptors, sensitizes GABAergic synapses to the CB1 receptor mediated presynaptic inhibitory action of exo- and endocannabinoids at the striatum [128]. Though the detailed receptor mechanisms responsible for these observations remain unknown, they reinforce previous evidence [43] that chronic and acute blockade of adenosine receptors may lead to opposite changes in the homeostatic balance mediated by adenosine.

A1 receptors appear also to be involved in motor incoordination induced by cannabinoids and this may occur at the cerebellum since intracerebellar injection of an A1 selective antagonist attenuates the motor incoordination induced by CB1 agonists [44]. A reciprocal ability for heterologous desensitization of CB1 and A1 responses due to prolonged agonist exposure has also been reported [89, 142]. 

### 1.5. Within Adenosine A1 A2A and A3 Receptor

Co-immunoprecipitation, BRET and radiologand-binding techniques showed the existence of A1-A2A receptor heteromers, intermolecular cross-talk and intramembrane receptor-receptor interactions in co-transfected human embryonic kidney (HEK) cells [23]. It has been proposed [57] that the A1-A2A receptor heteromer provides a "concentration-dependent switch" mechanism by which low and high concentrations of synaptic adenosine produce opposite effects, namely on glutamate release. However, other factors such as the topographical arrangement of ecto-enzymes, transporters and receptors as well the neuronal firing frequency may also influence the A1 versus A2A receptor mediated actions at each synapse where both receptors co-localize [136].

Cross talk between A1 and A2A receptors was clearly documented at the hippocampus, where activation of A2A receptors attenuates the ability of A1 receptor agonists to inhibit excitability and synaptic transmission [36, 110]. An A2A receptor-mediated decrease in A1 receptor binding was also shown in hippocampal [99] and striatal [50] synaptosomes. A2A receptor-induced inhibition of A1 receptor binding does not occur in membrane fragments, which indicates that the cross talk between A1 and A2A receptors involves a diffusible second messenger. The A2A/A1 receptor cross talk might be related to PKC, rather than to the classical A2A receptor second messenger, the adenylate cyclase-cAMP-PKA pathway, because the interactions between A2A and A1 receptors are prevented by PKC inhibitors but not by PKA inhibitors [50, 99]. PKC activators, such as phorbol esters, mimic the ability of A2A receptor agonists to decrease A1 receptor binding [99]. Thus, in what respects their ability to inhibit A1-receptor-mediated responses, A2A receptors appear to behave similarly to the PLC-coupled metabotropic glutamate receptors and to the muscarinic acetylcholine receptors [165], i.e. through a phosphoinositides-PKC-dependent pathway. Activation of PKC inhibits presynaptic A1 receptors on motor nerve terminals without affecting the affinity of competitive receptor antagonists [139], suggesting that the target of PKC is not the ligand binding domain, but probably a locus related to G-protein coupling, the G protein itself, or both. 

Besides the A2A/A1 receptor interaction, which can be observed either using BRET, binding, or functional studies with selective agonists to both receptors, there are other ways through which A2A receptors activation can also induce a decrease in tonic A1 receptor-mediated synaptic inhibition. Thus, A2A receptors enhance adenosine transport through equilibrative nucleoside transporters (ENT) with consequent reduction in the availability of endogenous extracellular adenosine and therefore in tonic activation of A1 receptors [115]. As it occurs with A2A receptor-mediated inhibition of A1 receptor binding [99], the A2A receptor-induced enhancement of ENT activity is lost upon inhibition of PKC, but not of PKA, suggesting the involvement of the PLC pathway rather the adenylate cyclase/cAMP one [115]. 

While evaluating the evoked release of acetylcholine at different frequencies of stimulation from hippocampal slices, it became clear that the A2A receptor-mediated enhancement of ENTs activity plays a pivotal role in adjusting adenosine neuromodulation to different physiological needs [115]. Thus, at high frequency neuronal firing there is a predominant release of ATP and a predominant formation of adenosine from released ATP [35]. Therefore, extracellular adenosine concentrations exceed the intracellular ones and the gradient of adenosine concentrations across the plasma membrane will direct ENTs to take up adenosine. Since A2A receptors are concomitantly activated, the A2A receptor-induced enhancement of ENT activity leads to an enhancement of removal of adenosine from synaptic cleft, therefore, to a reduced tonic A1-receptor mediated inhibition of hippocampal acetylcholine release at high frequency firing rates [115]. This A2A receptor-mediated inhibition of tonic inhibitory adenosinergic tonus may add to the A2A receptor inhibition of A1 receptor activation (see above) and eventually shut down tonic inhibition of A1 receptors upon synaptic plasticity [41]. This will efficiently reinforce the enhanced firing rate of cholinergic afferents into the hippocampus, which are known to play a key role in the control of cognitive processes such as attention and memory [79]. Influences of A2A receptors upon interneurones may also affect hippocampal dependent cognitive processes through exacerbation of neuronal firing. Thus, GABAergic inhibitory neurones at the hippocampus receive cholinergic inputs through excitatory α7 nicotinic receptors (nAChRs); these are acutely depressed by the neurotrophin, Brain Derived Neurotrophic Factor (BDNF), an action that required co-activation of adenosine A2A receptors [55]. One may therefore speculate that adenosine and BDNF, released during high frequency neuronal firing, have double influence upon the flow of information at the hippocampus: 1) by promoting facilitation of glutamatergic transmission and 2) by promoting an inhibition of inhibitory circuits, either through inhibition of A1 influences upon excitatory transmission or through a reduced cholinergic excitation of interneurones. A2A receptor-induced enhancement of GABA release [33] and GABA reuptake [32] may also contribute to sharp inhibitory transmission at the hippocampus.

Evidence that endogenous activation of A2A receptors plays a pivotal role on associative learning and upon reinforcement of relevant hippocampal circuits in vivo, has been provided recently. Thus, mice injected with an antagonist of A2A receptors have a profound impairment of conditioning learning as well as of experimentally evoked LTP of CA3/CA1 synapses, recorded concomitantly [65]. 

Desensitization of striatal A1 receptors is accompanied by a time-dependent amplification of A2-receptor-mediated stimulation of adenylate cyclase [1], indicating that A1 receptors also control A2A receptor functioning. Reciprocal control of neurotransmitter release by presynaptic A1 and A2A receptors were clearly observed at motor nerve terminals where endogenous A1 receptor-mediated inhibitory responses are enhanced in the presence of A2-receptor antagonists, and endogenous A2A receptor-mediated excitatory responses are increased in the presence of A1-receptor antagonists [28], clearly showing that the net modulation by endogenous adenosine depends upon a balanced A1/A2A receptor activation. In some cases, however, facilitation of neurotransmitter release due to A2A receptor activation is prevented by A1 receptor blockade [99], a finding that may have at least 2 interpretations: 1) the excitation due to A2A receptor activation results from relief of the tonic A1 receptor mediated inhibition [99], 2) there is a close molecular interaction between A1 and A2A receptors [57], so that co-activation of the A1 receptor by endogenous adenosine is required to allow the A2A receptor response. 

A positive cooperativity between A1 and A2 receptors, which also requires concomitant activation of metabotropic glutamate receptors, was observed in cultured astrocytes [108].

A3 receptor activation attenuates the synaptic inhibitory actions of adenosine in the CA1 area of the hippocampus [53]. Because adenosine A3 receptors might couple to phospholipase C, and phospholipase-C-coupled receptors are able to inhibit A1-receptor-mediated responses (see above) it is possible that this A3-A1-receptor mediated interaction involves this transducing system, in a way similar to that described in relation to the A3-receptor-mediated inhibition of metabotropic receptor functioning [101].

**Fig. (4):**
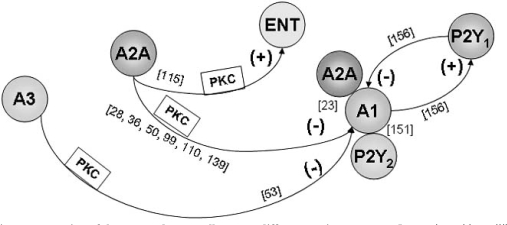
**Schematic representation of the reported cross-talk among different purine receptors.** Interaction with equilibrative nucleoside transporter (ENT) is also indicated. (+) denotes facilitation and (-) denotes inhibition. Evidence for tight molecular interactions, that may involve heteromerization, is also pointed out. See text and references (indicated in brackets) for details.

In summary, there are reciprocal interactions between different adenosine receptors (Fig. **4**). In neurons, A2A and A3 receptors attenuate A1 receptor functioning most probably through activation of a PKC-dependent pathway. A2A receptors also limit the availability of extracellular adenosine, by enhancing adenosine uptake, a process that also involves PKC. In astrocytes positive cooperation between A1 and A2A receptors might occur. 

### 1.6. Interaction with P2 Receptors

Although ATP and adenosine operate distinct families of receptors and play very distinct roles in the CNS (adenosine being exclusively a neuromodulator and ATP behaving as a neurotransmitter, neuromodulator, or co-modulator) interactions between receptors for these two ‘family related’ molecules have been reported. P2Y1 and A1 receptors can form heteromeric complexes and display a high degree of co-localization in the brain [170]. P2Y1 and A1 receptors are co-localized at glutamatergic synapses and surrounding astrocytes and P2Y1 receptor stimulation impairs the potency of A1 receptor coupling to G protein, whereas the stimulation of A1 receptors increases the functional responsiveness of Gq/11 coupled P2Y1 receptors [156]. Similarly, oligomerization of A1 and P2Y2 receptors generates a complex in which the simultaneous activation of the two receptors induces a structural alteration that interferes with signalling via G(i/o) but enhances signalling via G(q/11) [151]. 

The presynaptic facilitatory dinucleotide receptor is also under control by adenosine receptors co-localized in the same nerve terminals. Thus, the apparent affinity of diadenosine pentaphosphate (Ap5A) for its receptor in hippocampal nerve terminals is increased up to the low nanomolar range by co-activation of A1 or A2A receptors, whereas it is decreased towards the high micromolar range when A3 receptors are co-activated [46]. P2 receptor activation by endogenous ATP may also inhibit dinucleotide receptor functioning [47].

## 2. INTERACTION WITH IONOTROPIC RECEPTORS

Adenosine receptors interact with ionotropic receptors, and this has putative implications for neuroprotection, plasticity and learning, as it is the case of AMPA and NMDA glutamate receptors, as well as nicotinic acetylcholine receptors (nAChRs). Some of these interactions involve cyclic AMP – mediated ionotropic receptor phosphorylation followed by enhanced desensitization, others involve more complex transduction pathways.

### 2.1. Modulation of NMDA and AMPA Receptors by A1 and A2 Receptors

In isolated rat hippocampal neurones [42], as well as in bipolar retinal cells [31], A1 receptor activation inhibits NMDA-receptor-mediated currents. Interestingly, this inhibitory post-synaptic action of A1 receptor agonists is observed at very low concentrations, compatible with a tonic inhibitory action of adenosine. Accordingly, selective A1 receptor antagonism enhances the NMDA component of excitatory postsynaptic currents in CA1 hippocampal neurones, probably due to recruitment of previously silent NMDA receptors at synapses [88]. Endogenous adenosine, through a postsynaptic action inhibits voltage- and NMDA receptor-sensitive dendritic spikes in the CA1 area of the hippocampus [95]. Because of the important role played by NMDA receptors in synaptic plasticity phenomena it is conceivable that the ability of A1 receptors to inhibit NMDA receptor mediated currents, together with the well know A1 receptor mediated inhibition of glutamate release, are the basis of the A1-receptor-mediated inhibition of synaptic plasticity phenomena such as long-term potentiation (LTP) and long-term depression (LTD) at CA3/CA1 excitatory synapses of the hippocampus [41]. Similar mechanisms also contribute to A1-receptor-mediated neuroprotective actions during hypoxia [137] and to refrain epileptiform firing in CA1 pyramidal cells [95]. Interestingly, the expression of A1 receptors and of A1 mRNA transcripts is enhanced in astrocytes [9] and in neurones [10] by IL6, an interleukin whose expression is enhanced by A2B receptor activation in astrocytic cells [62]. Such regulatory loop leads to an enhancement of A1 receptor-mediated signalling under excitotoxic situations, such as hypoxia, excessive glutamate exposure or seizures, with a beneficial impact on neuronal survival [10]. 

ATP, probably by directly binding to the glutamate-binding pocket of the NR2B subunit of NMDA receptors can act as an inhibitor of NMDA receptors and attenuate NMDA-mediated neurotoxicity, an effect not mediated by ATP or adenosine receptors [112].

On medium spiny neurones at the striatum, A2A receptor activation inhibits (rather than facilitates) the conductance of NMDA receptor channels, by a mechanism involving the phospholipase C / inositol (1,4,5) triphosphate / calmodulin and calmodulin kinase II pathway [163]. In Mg(2+)-free conditions, therefore in conditions where NMDA receptors are not blocked, A2A receptor activation postsynaptically inhibits NMDA receptors in a subpopulation of striatal neurones; however, if NMDA receptors are blocked by Mg^2+^, the predominant A2A receptor mediated action is a presynaptic inhibition of GABA release [164]. Whether the A2A receptor mediated inhibition of NMDA receptors in the striatum explains the unexpected protective influence of A2A agonists towards NMDA-induced excitotoxicity [153] remains to be evaluated [117]. Other putative neuroprotective actions of A2A receptor agonists may involve a rather distinct mechanism, namely potentiation of the action of neurotrophic factors, such as glial derived neurotrophic factor (GDNF), on striatal dopaminergic nerve endings [74] and this may prove particularly useful in early stages of neurodegenetive diseases such as Parkinson’s disease, i.e. at stages where it may still be possible to rescue neurones from death through enhancement of trophic support.****

Long term potentiation (LTP) of synaptic transmission between CA3 and CA1 hippocampal areas of the hippocampus involves a postsynaptic facilitation of AMPA currents, a well known process that requires previous activation of NMDA receptors and involves both pre- (enhanced glutamate release) and post- (depolarization-induced relieve of NMDA receptor blockade by Mg^2+^) synaptic mechanisms. Interestingly, A2 receptor activation induces a form of LTP in the CA1 area that is NMDA receptor independent [87]. In contrast, A2A receptors localized postsynaptically at synapses between mossy fibres and CA3 pyramidal cells are essential for a form of LTP of NMDA currents, sparing AMPA currents [121]. Taking into consideration that CA3/CA1 LTP is predominantly NMDA receptor dependent, and that LTP at mossy fibres/CA3 synapses is predominantly pre-synaptic and NMDA receptor independent, it appears that A2A receptors are particularly devoted to unmask non-predominant forms of plasticity, therefore fine-tuning networking and information flow within the hippocampus. Whether NMDA receptors are required for the A2A receptor-dependent associative learning and concomitant CA3/CA1 synaptic plasticity in the hippocampus *in vivo* [65] awaits evaluation. 

In summary, the major trend for the interaction between ionotropic glutamate receptors and adenosine receptors is an A1-mediated inhibition and an A2A-mediated facilitation of NMDA receptor functioning. This fits with the global vocation of adenosine, with A1 receptors being inhibitory and neuroprotective, whereas A2A receptors facilitate synaptic reinforcement but also excitotoxicity phenomena. An NMDA receptor-induced exacerbation of A2A receptor mediated excitatory actions in the hippocampus [106], may reinforce positive interactions between these two receptors, closing a positive feedback loop where A2A and NMDA receptors reciprocally facilitate not only plasticity and learning but also neuronal damage. Interestingly NMDA receptors also exacerbate the ability of A1 receptors to increase G protein-activated inwardly rectifying K^+^ (GIRK) channel activation, a process critically involved in synaptic depotentiation [21].

### 2.2. Modulation of Nicotinic Acetylcholine Receptors

Endogenous adenosine, by activating A2A receptors coupled to the adenylate cyclase/cAMP transduction pathway, tonically downregulates presynaptic nicotinic acetylcholine receptors at either the skeletal neuromuscular junction [27] and myenteric plexus [52]. Other inhibitory influences of A2A receptors upon cholinergic receptors involve facilitation of BDNF-induced fast inhibition of α7 nAChR mediated currents, as shown at hippocampal interneurones [55].

## 3 INTERACTION WITH RECEPTORS FOR NEUROTROPHIC FACTORS

Receptors tyrosine kinase belong to a third class of membrane receptors, which by themselves possess catalytic activity, involving autophosphorylation in tyrosine residues as a consequence of ligand binding. This triggers a chain of phosphorylation events that lead to activation of several cascades that regulate cell death, survival and/or differentiation. Examples of this class of receptors are the receptors for neurotrophins, such as TrkA for Nerve Growth Factor (NGF), TrkB for BDNF, TrkC for Neurotrophin-3 (NT-3) or receptors for other theurotrophic factors, such as Ret for GDNF. 

Presynaptic depolarizationn which is known to increase extracellular adenosine levels, as well as enhancement of intracellular cyclic AMP, the most frequent A2 receptor transducing pathway, triggers synaptic actions of BDNF [12, 13]. On the other hand A2A receptors can transactivate TrkB receptors in the absence of the neurotrophin [94]. This transactivation requires long-term incubation with A2A receptor agonists and receptor internalization [120]. It is yet not clear whether TrkB receptor transactivation occurs through the same mechanism as the more recently identified ability of adenosine A2A receptors to trigger synaptic actions of BDNF. Indeed, it has been recently recognized that adenosine A2A receptor activation is a crucial requisite for the functioning of receptors for neurotrophic factors at synapses. This has been shown for the actions of BDNF on synaptic transmission [48, 49, 152], and LTP [66] at the CA1 area of the hippocampus as well as for the action of GDNF at striatal dopaminergic [74] and glutamatergic [73] nerve ending. Adenosine A2A and TrkB BDNF receptors can co-exist in the same nerve ending since the facilitatory action of adenosine A2A receptors upon TrkB-mediated BDNF action is also visible at the neuromuscular junction [118], a single nerve ending synapse model. The ability of BDNF to facilitate synaptic transmission is dependent of the age of the animals [48] and this may be related to the degree of activation of adenosine A2A receptors by endogenous adenosine at different ages. Thus, at infant rats, i.e. immediately after weaning, to trigger a BDNF facilitatory action it is necessary to increase the extracellular levels of adenosine, either by inhibiting adenosine kinase or by a brief depolarization [49, 118] or by inducing high frequency neuronal firing, such as those inducing LTP [66]; in all cases the actions of BDNF are lost by blocking A2A receptors with selective antagonists. In adult animals, BDNF per se, through TrkB receptor activation, can facilitate synaptic transmission but this effect is also fully lost upon blockade of adenosine A2A receptors [48] or in A2A receptor knockout mice [152]. Nicotinic α7 cholinergic currents in GABAergic hippocampal neurons are inhibited by BDNF, and this also requires co-activation of adenosine A2A receptors [55]. BDNF-induced inhibition of GABA transporters (GAT) of the predominant neural subtype, GAT1, does not fully depend upon co-activation of A2A receptors, since it is not abolished by A2A receptor blockade, but it may be enhanced by exogenous activation of A2A receptors [157]. Maximum transport velocity and surface expression of GAT1 is, however, directly affected by A2A receptors at GABAergic nerve terminals, through a mechanism that involves PKA-dependent relieve of PKC-induced GAT1 inhibition [32]. 

A2A receptors, due to their ability to enhance excitotoxicity fenomena, including glutamate release and action, are mostly regarded as promoters of neuronal death. However, in some cases, such as cultured retinal neurones, A2A receptors have been shown to protect neurones against glutamate induced excitotoxicity [61]. Whether this is due to the ability of A2A receptors to facilitate actions of neurotrophic factors, as it has been shown to occur in relation to A2A receptor-mediated neuroprotection of motor neurones [160] requires further investigation. It is worthwhile to note that while TrkB enhances survival of injured facial motor neurons in vivo [160], TrkB receptor activation by BDNF may render spinal cord cultured motor neurons more vulnerable to insult [103]. Interestingly enough, in both cases activation of A2A receptors by endogenous adenosine is required since A2A receptor antagonism prevents both the favourable [160] and the deleterious [103] TrkB mediated actions. 

A2A receptors activation enhances NGF-induced neurite outgrowth in PC12 cells and rescues NGF-induced neurite outgrowth impaired by blockade of the MAPK cascade, an action that requires PKA activation [19]. Furthermore A2A receptors activation, through Trk-dependent and phosphatidylinositol 3-kinase/Akt-mechanisms, promote PC12 cell survival after NGF withdrawal [94]. A similar A2A receptor-mediated neuroprotection mechanism has been shown to occur in hippocampal neurones after BDNF withdrawal [94]. Contrasting with A2A receptors which usually promote actions of neurotrophic factors, A1 receptors inhibit neurite outgrowth of cultured dorsal root ganglion neurons, both in the absence and in the presence of NGF [154].

Besides interactions at the receptor level, adenosine receptor activation may also induce release of neurotrophic factors. Thus, the expression and/or release of NGF are enhanced by activation of A2A receptors in microglia [80] and by activation of A1 receptors in astrocytes [22]. A2B receptors in astrocytes are also able to enhance GDNF expression [166]. A2A receptors are required for normal BDNF levels in the whole hippocampus [152].

Interactions among purinergic, growth factors, and cytokine signalling, are also highly relevant in non-pathologic brain functioning namely, to regulate neuron and glia maturation as well as development. Both ATP and adenosine receptors are involved in neuronal-dependent glia maturation [63]. The extracellular adenosine levels attained during high frequency neuronal firing are sufficient to stimulate adenosine receptors in olygodendrocyte ancestor cells, inhibiting their proliferation and stimulating their differentiation into myelinating oligodendrocytes [149]. Unfortunately, the nature of the adenosine receptor involved in these actions was not identified. In premyelinating Schwann cells, A2A receptors activate phosphorylation of ERK1/2 and inhibit Schwann cell proliferation without arresting differentiation [148]. 

### 3.1. Consequences of A2A/TrkB Cross Talk for Synaptic Plasticity

BDNF has an important role upon synaptic plasticity even in the adult hippocampus [102]. BDNF expression and release [7, 78], as well as release of adenosine [114], or of its precursor ATP [159] is more pronounced upon depolarization and during physiologically relevant patterns of neuronal activity, namely those that induce hippocampal LTP. Accordingly, released ATP [35] and high-frequency neuronal stimulation [28] favours A2A receptor activation. Therefore, high neuronal activity seems to create ideal physiological conditions for the interplay between adenosine A2A and TrkB receptors to occur. The finding that the facilitatory action of BDNF upon LTP in the CA1 area of the hippocampus is fully lost upon blockade of adenosine A2A receptors as well as upon depletion of extracellular adenosine [66] highlights the A2A receptor as a new physiologic partner, to the TrkB signalling processes that influences synaptic plasticity phenomena. Remarkably, associative learning and concomitant LTP recorded *in vivo* is also abolished in mice under the influence of a selective A2A receptor antagonist [65].

Another way A2A receptors have to influence BDNF-related plasticity is through the interplay with the homopentameric α-7 subtype of nAChR, which is particularly relevant for transmitter release and plasticity [75, 83] due to its high calcium permeability. Adenosine, through A2A receptors, and BDNF, through TrkB receptors, exert double control over α-7-nicotinic currents at GABAergic interneurons in the hippocampus, as it can be concluded from the finding that blockade of A2A receptors abolishes the BDNF-induced current inhibition [55]. Since postsynaptic α7 nAChR-mediated inputs to GABAergic interneurons regulate inhibition within the hippocampus, A2A receptors by allowing the inhibition of cholinergic currents by BDNF, might temporarily relieve GABAergic inhibition, therefore facilitating plasticity phenomena. 

### 3.2. Pathophysiological Implications of the Cross-Talk Between Adenosine and Neurotrophic Factors

A decrease in levels and/or action of neurotrophic factors have been implicated in the pathophysiological mechanisms of many diseases of the nervous system, such as Alzheimer’s disease, Parkinson’s disease, Huntington’s disease, diabetic neuropathies, amyotrophic lateral sclerosis and even depression, therefore making the use of the naturally occurring neurotrophic factors very promising for treatment of these disorders [17, 134]. However, until now the pharmacological administration of neurotrophic factors in vivo has not been easy because these molecules are unable to cross the blood brain barrier, making invasive application strategies like intracerebroventricular infusion necessary. The evidence that adenosine A2A receptors trigger or facilitate actions of neurotrophins upon synaptic strength and neuronal survival highlights interest upon the use of adenosine A2A receptor agonists that cross the blood brain barrier as tools to potentiate neurotrophic actions in the brain. The expression [34] and functioning [122] of A2A receptors in the forebrain increases with age, whereas the number of TrkB receptors is markedly lower in the hippocampus of aged rats [144]. The increase in the adenosine A2A receptor tonus partially compensates the loss of TrkB receptors upon ageing, rescuing to certain degree the facilitatory action of BDNF in aged animals [48]. This might prove particularly important in the prevention of neurodegeneration, since neurodegenerative diseases are most frequent upon ageing. Furthermore, it reinforces the therapeutic potential of adenosine-related therapies to influence the actions of neurotrophic factors in old subjects. 

A promising area of research, as regards adenosine/BDNF cross talk, is Huntington’s disease, which has been associated with low BDNF levels in the cortical-striatal pathway, most probably due to a loss of function of mutated huntingtin [174]. How the low BDNF signalling can be compensated by A2A receptor activation deserves detailed investigation. Interestingly, daily administration of the A2A receptor agonist, CGS 21680, delays progressive deterioration of motor performance, huntingtin aggregation and increase in striatal choline levels in a transgenic mouse model (R6/2) of Huntington’s disease [20]. This animal model involves genetic mutation of Huntingtin, therefore most probably, a reduction of striatal BDNF levels since there is strong evidence that a major contributing pathway to striatal degeneration in Huntington’s disease is an impairment of anterograde transport BDNF from the cortex to the striatum [68,150]. 

A particular mention has to be made to epilepsy, where neurotrophic factors have been considered both harmful, being causal mediators in the development of acquired epileptic syndromes, and eventually useful to attenuate epilepsy-associated neuronal damage [131, 146]. On the top of this controversy we can add discrepant findings of both anticonvulsive [81] and pro-convulsive [171] adenosine A2A receptor-mediated actions, the pro-convulsive being much more expected due to the usually excitatory nature of these receptors. A better understanding of the influence of adenosine A2A receptors upon the actions of BDNF in epilepsy is particularly relevant because therapies that lead to localized enhancement of extracellular adenosine levels (adenosine augmentation therapies, AATs) are currently under development [161] as a strategy to fight pharmacoresistant epilepsy, but research in this area has been only focusing on the beneficial influences mediated by inhibitory adenosine A1 receptors [11]. Highly promising results with AATs were already obtained in animal models, where intrahippocampal implants of stem cells engineered to lack adenosine kinase (therefore locally releasing considerable amounts of adenosine into the extracellular space) prevent epileptogenesis [96]. Once in the extracellular space, adenosine may, however, reach not only the predominantly expressed adenosine receptors in the forebrain, the A1 receptors, but also the A2A receptors, which in spite of their low density in the forebrain have high affinity for adenosine and, as reviewed above, are mostly devoted to fine tune neuronal activity. It may happen that A2A receptors prove detrimental in epilepsy due to their ability to enhance excitability, and therefore these receptors need to be concomitantly blocked in adenosine augmentation therapies; conversely, it may happen that these receptors facilitate the positive influences of BDNF upon neuronal survival and therefore, an A2A receptor mediated favourable influence would add to the A1 receptor mediated one. Lastly, it may happen that these receptors due to its low density are without any influence upon the outcome of AATs in epilepsy.

Results from clinical and basic studies have demonstrated that stress and depression decrease BDNF expression and neurogenesis, leading to the neurotrophic hypothesis of depression [17, 90]. The involvement of TrkB receptors upon sensitivity to antidepressive treatment has recently been highlighted [97]. As A2A receptor activation may have anti-depressive action [85], one may, therefore, speculate that the ability of A2A receptors to facilitation the actions BDNF may contribute to the antidepressive actions of adenosine. It is worthwhile to note that deep brain stimulation, now widely used by neurosurgeons to treat tremor and other movement disorders, as well as in a number of psychiatric diseases, including obsessive-compulsive disorders and depression [92], produces its effects by inducing the release of ATP which is subsequently converted extracellularly to adenosine [8]. Whether adenosine, through facilitation of BDNF actions, contributes to the antidepresssive properties of deep brain stimulation, also awaits further evaluation.

**Fig. (5):**
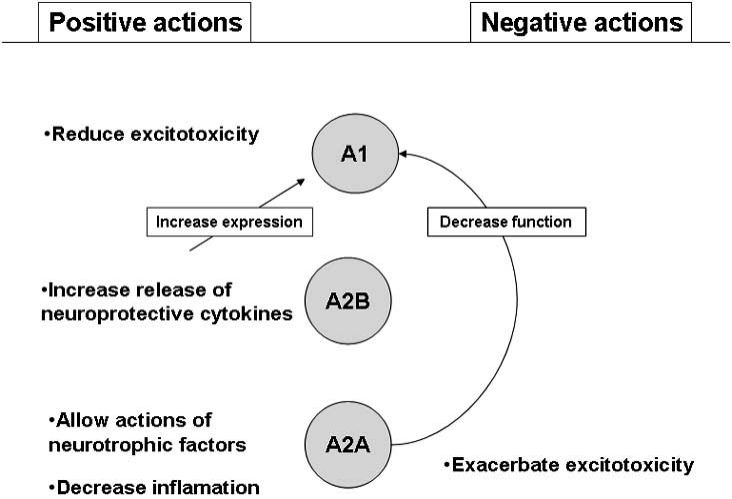
**Negative and positive actions of A1 and A2A adenosine receptors to protect neuronal cells.** Note that whereas A1 receptors possess predominant neuroprotective actions, A2A receptors may operate mechanisms leading to neuronal protection or damage. A better knowledge of the time windows for those actions, and how to manipulate them will allow the increase in the therapeutic potential of adenosine related drugs.

Finally, the cross-talk between adenosine A2A receptors and receptors for neurotrophins points to the need of caution about therapies with A2A receptor antagonists in neurodegenerative diseases (see Fig. **5**), as it has been proposed for Parkinson’s disease to ameliorate L-DOPA induced dyskinesias [104]. Indeed, the identification of postsynaptic A2A/D2 receptor interactions in the striatum together with the findings that A2A receptor antagonists are neuroprotective in Parkinson’s disease models [18] and increase dopamine synthesis from L-DOPA [72], led to the proposal for the use of A2A receptor antagonists in Parkinson´s disease. On the other hand, neurotrophic factors, in particular GDNF, may be a potential therapeutic approach in the management of Parkinson´s disease [100, 113]. GDNF control of the glutamatergic cortico-striatal pathway requires tonic activation of adenosine A2A receptors [73]. Also, the faciliatory action of GDNF upon dopamine release in the striatum is lost upon blockade of A2A receptors and is enhanced by A2A receptor agonists [74]. All these observations point towards the need for further studies on the consequences of long-term therapy with A2A receptor blockers in neurodegenerative diseases, where neurotrophic factors may play a beneficial role. One issue that should be explored in the future is the optimal time window for combined beneficial effects for neurotrophic factors and adenosine A2A receptor agonists/antagonists. Perhaps, in the late stages of neurodegenerative diseases, A2A receptor antagonists may be advantageous to prevent and/or attenuate dyskinesias; however, in the early stages, where neurones are struggling for life and an enhancement of neurotrophic factors is highly desirable, A2A receptor antagonists should be avoided and A2A agonists could be considered to potentiate neurotrophic influences. 

## CONCLUDING REMARKS

Data presently available allow envisaging adenosine as a sort of ‘universal modulator’ or a ‘maestro’, which, via controlling the release and action of many synaptic mediators, would serve as the main molecule involved in the coordination of brain activity. A better understanding of the intimate cross-talk that this modulator establishes with other signaling molecules will enhance our understanding of brain function and dysfunction, such as cognition, neurodegenerative diseases, psychiatric diseases, and drug addiction.
